# Laparoscopic versus open wedge resection for gastrointestinal stromal tumors of the stomach: a single-center 8-year retrospective cohort study of 156 patients with long-term follow-up

**DOI:** 10.1186/s12893-015-0040-2

**Published:** 2015-05-09

**Authors:** Jia-Qin Cai, Ke Chen, Yi-Ping Mou, Yu Pan, Xiao-Wu Xu, Yu-Cheng Zhou, Chao-Jie Huang

**Affiliations:** Department of General Surgery, Sir Run Run Shaw Hospital, School of Medicine, Zhejiang University, 3 East Qingchun Road, Hangzhou, 310016 Zhejiang Province China

**Keywords:** Gastrointestinal stromal tumor, Laparoscopy, Meta-analysis, Survival

## Abstract

**Background:**

The aim of this study was to compared laparoscopic (LWR) and open wedge resection (OWR) for the treatment of gastric gastrointestinal stromal tumors (GISTs).

**Methods:**

The data of 156 consecutive GISTs patients underwent LWR or OWR between January 2006 and December 2013 were collected retrospectively. The surgical outcomes and the long-term survival rates were compared. Besides, a rapid systematic review and meta-analysis were conducted.

**Results:**

Clinicopathological characteristics of the patients were similar between the two groups. The LWR group was associated with less intraoperative blood loss (67.3 vs. 142.7 ml, *P* < 0.001), earlier postoperative flatus (2.3 vs. 3.2 days, *P* < 0.001), earlier oral intake (3.2 vs. 4.1 days, *P* < 0.001) and shorter postoperative hospital stay (6.0 vs. 8.0 days, *P* = 0.001). The incidence of postoperative complications was lower in LWR group but did not reach statistical significance (4/90, 4.4% vs. 8/66, 12.1%, *P* = 0.12). No significant difference was observed in 3-year relapse-free survival rate between the two groups (98.6% vs. 96.4%, *P* > 0.05). The meta-analysis revealed similar results except less overall complications in the LWR group (RR = 0.49, 95% CI, 0.25 to 0.95, *P* = 0.04). And the recurrence risk was similar in two group (RR = 0.80, 95% CI, 0.28 to 2.27, *P* > 0.05).

**Conclusions:**

LWR is a technically and oncologically safe and feasible approach for gastric GISTs compared with OWR. Moreover, LWR appears to be a preferable choice with mini-invasive benefits.

## Background

Gastrointestinal stromal tumors (GISTs), the most common mesenchymal tumor of the gut, are often characterized by high expression of KIT [1,2]. The most common sites for GIST include the stomach (60%) and jejunum or ileum (30%) followed by duodenum (5%), colon and rectum (less than 5%), esophagus (less than 1%), and appendix (less than 1%) [2]. GISTs have malignant potential, and it is reported that recurrence of GISTs often occured at the peritoneal surface or liver [3]. Surgical resection is the mainstay management for primary localized GISTs. As submucosal and lymphatic spread are rare, the surgical principles are composed of an R0 resection with a normal mucosa margin, no systemic lymph node dissection, and avoidance of perforation, which results in peritoneal seeding even in cases with otherwise low risk profiles [2-4].

Since the development of minimally invasive surgical approaches, laparoscopic surgery for gastrointestinal tumors has evolved rapidly over the past decade. Various types of laparoscopic approaches for GISTs have been described, including wedge resection of the stomach, intragastric tumor resection, and combined endoscopic-laparoscopic resection [5-8]. For gastric GISTs, lymph node metastases are rare and localised resection with a clear margin of 1 to 2 cm appears to be an adequate treatment [9,10]. Besides, recent evidence has shown that survival depends on the tumor size and histological features rather than the extent of resection [3]. Therefore, gastric GISTs can be treated without major anatomical resections [11] and are suitable for laparoscopic wedge resection (LWR). Several case series have proved the safety and feasibility of LWR for gastric GISTs, however, the oncologic benefits of LWR have not been widely reported and the sample size of those researches were relatively small. In the current study, we retrospectively reviewed data for GIST patients who underwent LWR and traditional open wedge resection (OWR) at our hospital between 2006 and 2013. The clinical data, benefits of operation, perioperative outcomes, and oncologic outcomes were reviewed. Besides, a rapid systematic review with a meta-analysis was conducted to further assess accurately the current status of LWR for gastric GIST.

## Methods

### Patients

Between January 2006 and December 2013, 177 consecutive patients with suspected gastric GIST underwent laparoscopic or open wedge resection in the Department of General Surgery at the Sir Run Run Shaw Hospital, China. The exclusion criteria included: (1) patients concomitant with tumors outside stomach; (2) patients with metastatic disease at the time of operation; (3) patients diagnosed as other types of submucosal tumor after immunohistochemical examination. Blood tests, chest X-rays, enhanced computed tomography scans of the abdomen and pelvis, and endoscopic ultrasonography were performed before operation. This study protocol was prospectively approved by ethics committee of Sir Run Run Shaw Hospital, School of Medicine, Zhejiang University and conducted in accordance with the ethical guidelines of the Declaration of Helsinki. Informed consent was signed prior to surgery by each case.

### Surgical procedure

The patient is placed in the supine position under general anesthesia. The surgeon stood on the right side of the patient. One assistant stood on the right side of the patient and held the laparoscope, and another stood on the left side of the patient. Carbon dioxide pneumoperitoneum was established through the Veress needle and set at 15 mmHg. One initial 10-mm trocar was inserted for laparoscopy below the umbilicus and another four trocars (one of 12 mm, three of 5 mm) were inserted into the left upper flank, left flank, right upper flank, and right flank quadrants; a total of five trocars were inserted, and arranged in a V-shape.

Mobilizing the tumor before excised were usually as fellows: Tumor in anterior wall of the gastric body and pylorus was excised directly. If tumor was in anterior wall near lesser curvature, the hepatogastric ligament was dissected firstly to free it. If it was in anterior wall near great curvature, parts of gastrocolic ligament and gastrosplenic ligament were dissected firstly. For tumor located in posterior wall, the gastrocolic and gastrosplenic ligament were dissected, then lifted up the stomach to expose the tumor. Those in fundus, the gastrocolic and gastrosplenic ligament was also dissected as well as left gastroepiploic vessels and short gastric vessels, so the fundus can be mobilized and the tumor can be expose. Gastroscopy was used intraoperatively to evaluate tumor localization if necessary. Tumor was excised using ultrasonic scalpel or endoscopic linear stapler with at least 1-2 cm surgical margin. The defect left by excision using ultrasonic scalpel in the gastric wall was reinforced using laparoscopic hand-suturing technique. If the tumors were near the cardia or pylorus, excision using ultrasonic scalpel was preferred, as it can reduce the risk of cardiac or pyloric stricture. While the tumors were in the gastric fundus, excision using endoscopic linear stapler was preferred, for tumor had a good mobility to perform this procedure easily. For tumors located near the esophagogastric junction, especially those with intraluminal growth, we used laparoscopic transgastric wedge resection to avoid deformity or stenosis in the gastric inlet. A summary of the detailed transgastric resection were described in our published article [6].

### Data collection and follow-up evaluation

The patients’ demographic data, surgical outcomes, and complications were reviewed, and the survival rate was analyzed. The prognostic indicators of GISTs were based on tumor size and mitotic index, according to the risk assessment classification proposed by Fletcher et al. [12] Gastric GISTs were categorized for malignant potential as very low risk (<2 cm and <5 mitoses/50 high-power fields, HPFs), low risk (2--5 cm and <5 mitoses/50 HPFs), intermediate risk (<5 cm and 6--10 mitoses/50 HPFs or 5--10 cm and <5 mitoses/50 HPFs), and high risk (>5 cm and >5 mitoses/50 HPFs, >10 cm and any mitotic rate, any size, or >10 mitoses/50 HPFs). The immunohistochemical analysis included detection of CD117, CD34, smooth muscle actin protein (SMA), S-100, and desmin expression. Follow-up results were obtained from patients’ medical records and telephone calls, and recurrence was determined by endoscopy, computed tomography, positron emission tomography, etc, and the last follow-up day was January 30, 2014.

### A rapid systematic review and meta-analysis

We searched PubMed, Cochrane Library, Web of Science and BIOSIS Previews for literature comparing LWR and OWR published between January 1995 and April 2014. The following keywords were used: “gastrointestinal stromal tumor”, “GIST”, “laparoscopy”, “laparoscopic”, “minimally invasive surgery”, “gastric resection”, “gastric surgery”, and “comparative study”. The language of the publications was confined to English. The papers containing any of the following were excluded: (1) concomitant with tumors outside stomach; (2) not wedge resection; (3) if there was overlap between authors or centers, the higher quality or more recent literature were selected. Two investigators reviewed the titles and abstracts, and assessed the full text to establish eligibility. The Newcastle-Ottawa Quality Assessment Scale (NOS) was used for quality assessment of observational studies. A threshold of six stars or above has been considered indicative of high quality.

### Statistical analysis

Quantitative data are given as the means ± standard deviations (SDs). The differences in the measurement data were compared using the Student’s *t* test, and comparisons between groups were tested using the *χ*^2^ test or the Fisher exact probability test. Relapse-free survival (RFS) rates were calculated by the Kaplan-Meier method using SPSS software, version 18.0 (SPSS Inc, Chicago, United States). Relapse-free survival was calculated from the day of surgery to the day of recurrence. *P* < 0.05 was considered statistically significant.

The meta-analysis was performed in line with recommendations from the Cochrane Collaboration and the Quality of Reporting of Meta-Analyses guidelines [13,14]. Continuous variables were assessed using the weighted mean difference (WMD), and dichotomous variables were analyzed using the risk ratio (RR). If the study provided medians and ranges instead of means and standard deviations (SDs), we estimated the means and SDs as described by Hozo et al. [15]. To account for clinical heterogeneity, which refers to diversity in a sense that is relevant for clinical situations, we used the random effects model based on DerSimonian and Laird’s method. Potential publication bias was determined by conducting informal visual inspection of funnel plots based on the complications. Data analyses were performed using Review Manage version 5.1 (RevMan 5.1) software downloaded from the Cochrane Library. *P* < 0.05 was considered statistically significant.

## Results

### Demographic and clinicopathologic characteristics

Among the 177 patients, 21 were excluded. Five patients with coexistence of any other malignancies were excluded. Fourteen patients were excluded because diagnosed as other types of submucosal tumor instead of GIST. Two patients with metastatic disease were also excluded. Finally, 156 patients were enrolled into this study. Among them, 90 patients underwent laparoscopic wedge resection (LWR group) for gastric GISTs, while 66 patients received open wedge resection (OWR group).

The LWR group included 31 males (34.4%), and the mean age was 58.6 ± 10.7 years. The OWR group included 29 males (43.9%), and the mean age was 56.8 ± 11.9 years. Mean body mass index (BMI) for the LWR was 22.8 ± 3.1 kg/m2 compared with 23.3 ± 3.7 kg/m2 among the OWR. The ASA score for each patient was: ASA I [LWR, 44 (48.9%); OWR, 33 (50.0%)], ASA II [LWR, 41 (45.6%); OWR, 30 (45.5%)], and ASA III [LWR, 5 (5.6%); OWR, 3 (4.5%)]. No statistical differences were observed between the group’s demographic characteristics, ASA scores, comorbidities, and BMI (Table 1). The mean preoperative hemoglobin and albumin levels were 12.8 ± 1.9 g/dL and 42.2 ± 4.2 g/L in LWR group, and 12.7 ± 2.4 g/dL and 42.9 ± 4.5 g/L in OWR group.

**Table 1:** **Clinical characteristics of patients**

**Variable(%)**	**LWR(n = 90)**	**OWR(n = 66)**	***P*** **value**
Gender (male/female)	31/59	29/37	0.79
Age (years)	58.6 ± 10.7	56.8 ± 11.9	0.33
BMI (kg/m2)	22.8 ± 3.1	23.3 ± 3.7	0.29
ASA classification (I/II/III)	44/41/5	33/30/3	1.00
Comorbidities (yes)	35(38.9)	26(39.4)	0.77
Hypertension	29(32.2)	17(25.8)	
Diabetes mellitus	9(10)	7(10.6)	
Cardiovascular	5(5.6)	4(6.1)	
Pulmonary	2(2.2)	1(1.5)	
Previous abdominal surgery	22(24.4)	20(30.3)	0.42
Preoperative hemoglobin	12.8 ± 1.9	12.7 ± 2.4	0.91
Preoperative albumin	42.2 ± 4.2	42.9 ± 4.5	0.31

The pathological variables of the patients are summarized in Table 2. The mean tumor size in the LWR group was 3.5 cm and in the OWR group it was 4.3 cm. The mean tumor size in the LWR group was smaller than OWR group (P = 0.02). However, for properties, there was no statistically significant difference between the two groups according to Fletcher’s criteria (*P* > 0.05). In all GIST patients, 84.6% had a mitotic rate of fewer than 5 mitoses per 50 high-power field (HPF), 9.6% had a mitotic rate between 5 and 10 mitoses per 50 HPF, and 5.8% had more than 10 mitoses per 50 HPF. The two groups were comparable with respect to tumor location, with the majority of patients having tumors located in the gastric body or fundus (77.7% in the LWR and 63.6% in the OWR group).

**Table 2:** **Pathologic features of patients**

**Variable(%)**	**LWR(n = 90)**	**OWR(n = 66)**	***P*** **value**
Tumor size (cm)	3.5 ± 1.9	4.3 ± 2.4	0.02
Tumor location			0.08
Cardia	14(15.6)	10(15.2)	
Fundus	29(32.2)	16(24.2)	
Body near lesser curvature	11(12.2)	4(6.1)	
Body near greater curvature	30(33.3)	22(33.3)	
Antrum	6(6.7)	14(21.2)	
Mitotic rate (per 50 HPF)			0.20
<5	80(88.9)	52(78.8)	
5 ~ 10	7(7.8)	8(12.1)	
>10	3(3.3)	6(9.1)	
Immunohistochemistry			
CD117(+)	86(95.6)	66(100)	0.11
CD34(+)	87(96.7)	63(95.5)	0.20
DOG-1(+)	72(80.0)	58(87.9)	0.19
SMA	30(33.3)	17(25.8)	0.31
S-100	16(17.8)	14(21.2)	0.59
Desmin	9(10)	6(9.1)	0.95
Fletcher classification			0.51
Very low risk	20(22.2)	13(19.7)	
Low risk	46(51.1)	28(42.4)	
Intermediate risk	16(17.8)	16(24.2)	
High risk	8(8.9)	9(13.6)	

### Operative outcomes and postoperative recovery

The outcomes associated with surgery and postoperative recovery are shown in Table 3. In the OWR group, the mean amount of estimated intraoperative bleeding was more than in the LWR (67.3 ± 80.5 ml vs. 142.7 ± 102.0; *P* < 0.01). Mean operative time was similar between groups (106.6 ± 40.1 min vs. 119.9 ± 59.9; *P* > 0.05). There were 21 cases in the LWR group used intraoperative endoscopy to locate the tumors.

**Table 3:** **Operative findings and postoperative clinical courses**

**Variable**	**LWR(n = 90)**	**OWR(n = 66)**	***P*** **value**
Operation time (min)	106.6 ± 40.1	119.9 ± 59.9	0.12
Blood loss (ml)	67.3 ± 80.5	142.7 ± 102.0	0.000
Intraoperative endoscopy	21(23.3)	0(0.0)	
Time to first flatus (days)	2.3 ± 0.9	3.2 ± 0.8	0.000
Time to oral intake (days)	3.2 ± 1.0	4.1 ± 0.9	0.000
Postoperative hospital stay (days)	6.0 ± 2.1	8.0 ± 5.1	0.001
Postoperative complications	4(4.4)	8(12.1)	0.08
Anastomotic hemorrhage	0	2	
Abdominal abscess	0	1	
Delayed gastric emptying	3	3	
Wound infection	0	2	
Pulmonary infection	1	0	

Mean times to postoperative flatus and oral intake were significantly shorter in the LWR group than in the OWR group (2.3 days vs. 3.2 days, *P* < 0.01, and 3.2 days versus 4.1 days, *P* < 0.01). The mean duration of postoperative hospital stay was two days longer in the OWR group (6.0 days vs. 8.0 days, *P* < 0.01).

The incidence of postoperative complications was higher for the OWR group than the LWR group. But the difference did not reach statistical significance (4.4% vs. 12.1%, *P* = 0.08). Incidences of morbidity in LWR group included three cases of delayed gastric emptying and one case of pulmonary infection. Complications in OWR group included two cases of anastomotic hemorrhage, one case of abdominal abscess, three cases of delayed gastric emptying and two cases of wound infection. All these complications were controlled with conservative treatment.

### Follow-up results

Of the 156 identified patients, 149 (95.5%) were followed up and 7 were lost to follow-up. Follow-up data were available for 87 (96.6%) and 62 (93.9%) of patients treated with LWR and OWR, respectively. The median follow-up was 21.0 months (range, 1-90 months) in the LWR group and 44.5 months (range, 1-96 months) in the OWR group.

**Figure 1:**
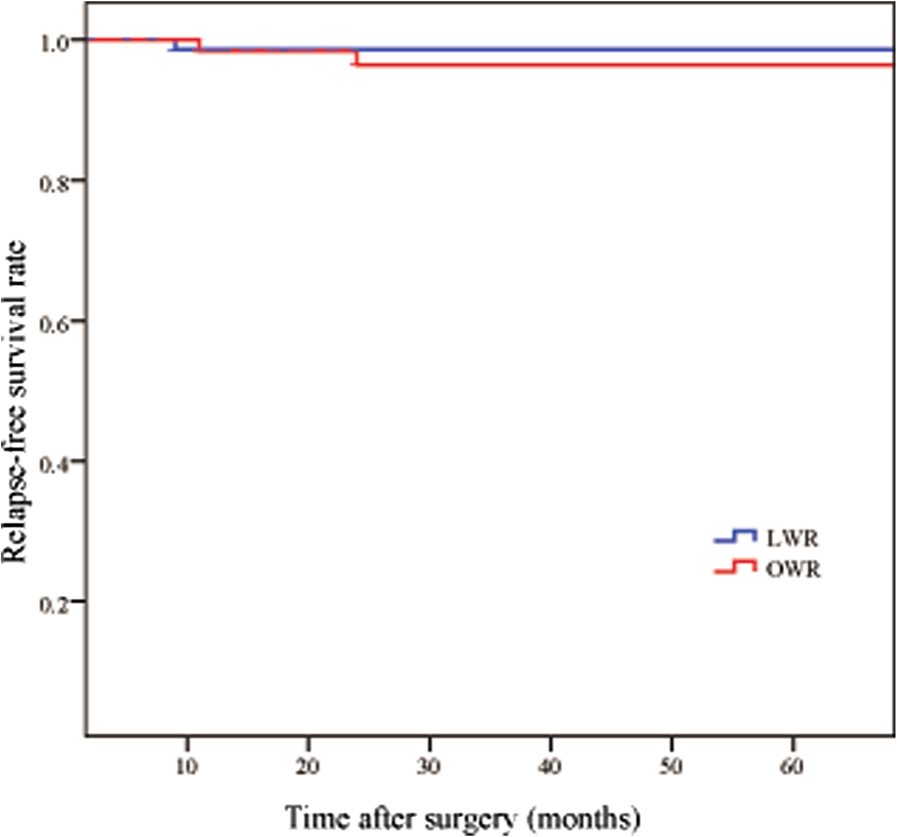
Kaplan-Meier relapse-free survival curves of patients underwent laparoscopic (LWR) or open wedge resection (OWR) gastric GISTs.

One patient in the LWR group diagnosed with low risk of disease recurrence developed metachronous liver metastasis 9 months after operation. Two patients in the OWR group developed liver metastasis 11 months and 24 months after operation, respectively. They were both diagnosed with high risk of recurrence and were still alive at the end of last follow-up. One patient of low risk of recurrence in the LWR group dead of breast cancer 42 months after gastric surgery. However, there was no evidence of GIST recurrence before her death. The 3-year RFS rates was 98.6% in LWR group and 96.4% in OWR group. There were no significant differences between the two groups (*P* > 0.05) (Figure 1).

### A rapid systematic review and meta-analysis

**Figure 2:**
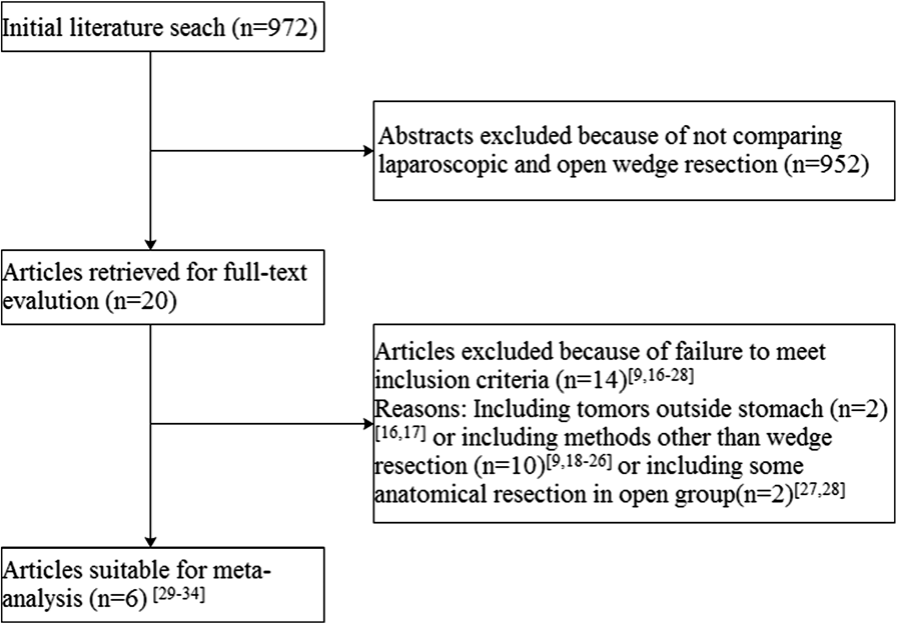
Flow chart of literature search strategies.

The initial search strategy retrieved 972 publications in English. After the titles and abstracts were reviewed, papers without comparison of LWR and OWR were excluded, which left 20 comparative studies, fourteen [9,16-28] of which did not meet the inclusion criteria and were excluded. This left a total of six comparative observational studies [29-34]. A flow chart of the search strategies is illustrated in Figure 2. Including the present data, a total of 525 patients were included in the analysis with 264 undergoing LWR (50.3%) and 261 undergoing OWR (49.7%). According to the NOS, one out of the six observational studies got 7 stars, two articles got 8 stars, and the remaining three got 9 stars. The characteristics and methodological quality assessment scores of the included studies are shown in Table 4.

**Table 4:** **Summary of studies included in the meta-analysis**

**Author**	**Nation**	**Study type**	**Publication year**	**Study period**	**Sample size**		**Follow-up (months)**		**Quality scores**
					**LWR**	**OWR**	**LWR**	**OWR**	
Ishikawa [29]	Japan	Retro	2006	1993-2004	14	7	60(5–119)	61(3–130)	8
Mochizuki [30]	Japan	Retro	2006	2000-2004	12	10	26 (6–53)	NR	8
Catena [31]	Italy	Pros	2008	1995-2006	21	25	35(5–58)	91(80–136)	9
Goh [32]	Singapore	Retro	2010	2001-2009	14	39	8(3–60)	21(2–72)	7
Lee [33]	Korea	Retro	2011	2001-2008	50	50	21(0–64)	22(0–93)	9
Wan [34]	China	Retro	2012	2004-2011	63	64	NR	NR	9

Retro: retrospective observational study; Pros: prospective observational study; NR: not reported.

**Figure 3:**
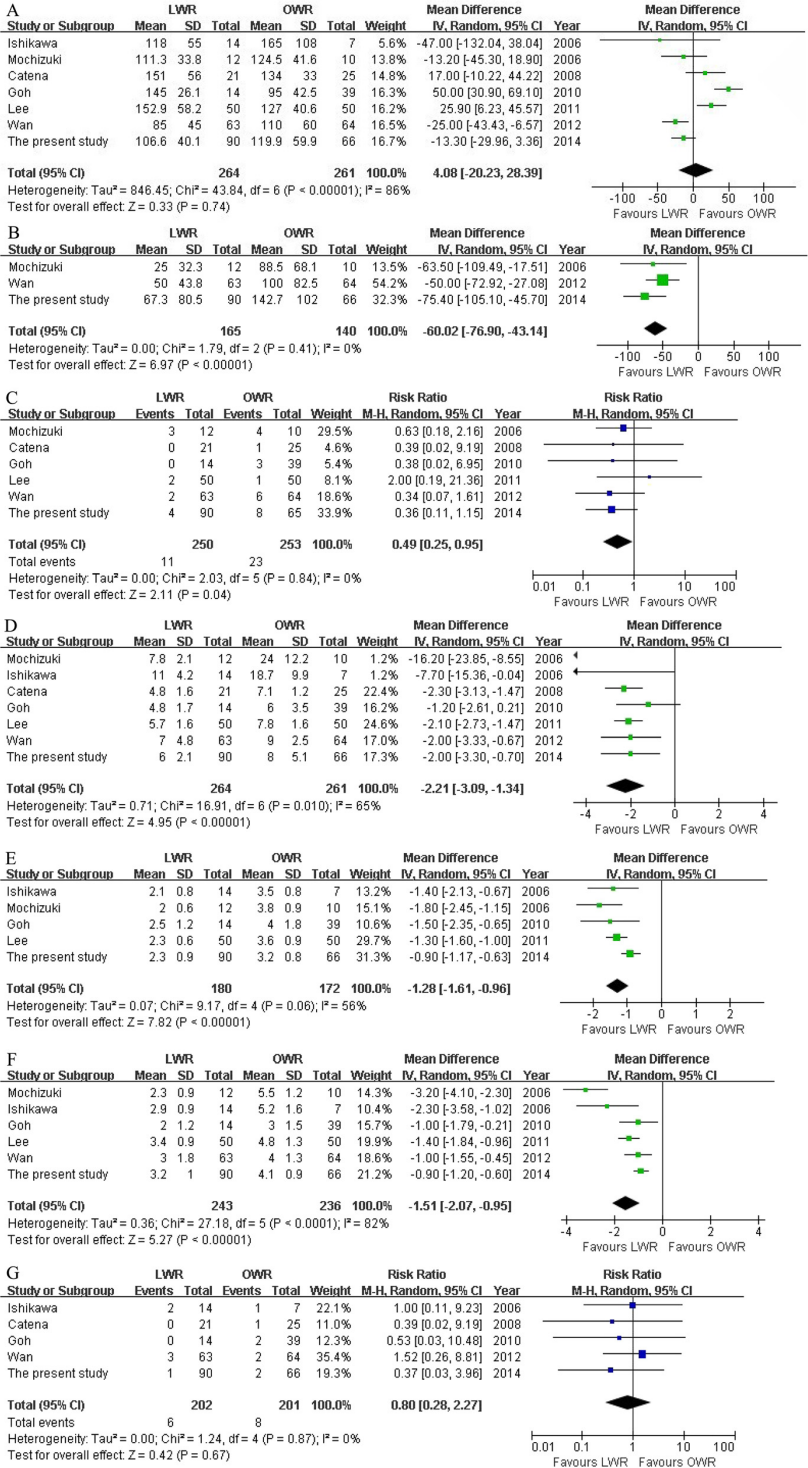
Meta-analysis of the pooled data. **(A)** Operative time. **(B)** Intraoperative blood loss. **(C)** Overall complications. **(D)** Postoperative hospital stay. **(E)** First flatus. **(F)** Oral intake. **(G)** recurrences.

**Figure 4:**
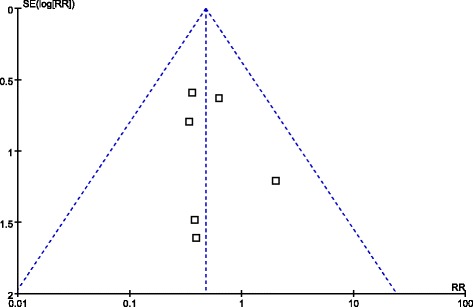
Funnel plot of the overall postoperative complications.

All studies reported operative time [29-34]. The present analysis showed no statistically significant difference in the operative time of the two groups (WMD = 4.08 min; 95% CI, -20.23 to 28.39; *P* = 0.74) (Figure 3A). Two studies reported blood loss [30,34]. Intraoperative blood loss was significantly lower in the LWR compared with the OWR group (WMD = -60.02 ml; 95% CI, -76.90 to -43.14 ml; *P* < 0.01) (Figure 3B). All studies reported overall complications [29-34]. There were significantly fewer overall complications in the LWR than the OWR group (RR = 0.49, 95% CI, 0.25 to 0.95, *P* = 0.04) (Figure 3C). Visual inspection of the funnel plot revealed symmetry, indicating no serious publication bias (Figure 4). Morbidity was specified in two studies [31,34]. One study reported a wound infection in OWR group [31]. Another reported one wound infection and one anastomosis site bleeding in LWR group and one wound infection, one wound dehiscence and four pyrexia in OWR group [34].

All studies reported duration of hospital stay [29-34]. Patients in the LWR group had a shorter postoperative hospital stay (WMD = -2.21 days; 95% CI, -3.09 to -1.34, *P* < 0.01) (Figure 3D). Four studies reported time to first flatus [29,30,32,33] and five studies reported time to oral intake [29,30,32-34]. Patients in the LWR group were able to pass flatus (WMD = -1.28 days; 95% CI, -1.61 to -0.96, *P* < 0.01) (Figure 3E) and resume oral intake earlier (WMD = -1.51 days; 95% CI, -2.07 to -0.95, *P* < 0.01) (Figure 3F).

During the follow-up period, recurrence was observed in four studies [29,31,32,34]. Including our study, the recurrence risk in LWR was 2.3% (6/264) and 3.1% (8/261) in OWR, but the difference between LWR and OWR was not significant (RR = 0.80, 95% CI, 0.28 to 2.27, *P* = 0.67) (Figure 3G). Wan et al. [34] have reported that there was no significant difference in the 5-year RFS between LWR and OWR (93.7% in LWR, 95.5% in OWR). Goh et al. [32] also have reported that there was no significant differences in RFS between groups.

## Discussion

Adenocarcinomas are the most common tumors of the stomach, whereas submucosal tumors of the stomach such as GISTs are rare. Unlike adenocarcinomas of gastrointestinal tract, GISTs showed that negative macroscopic margins only may portends a survival benefit [3]. Lymphatic spread is quite uncommon, and as such, systemic lymphadenectomy has been deemed unnecessary [2,3]. These characteristics, along with GISTs’ tendency to grow in an exophytic manner, led many surgeons prefer wedge resection rather than formal gastrectomy for gastric GISTs whenever feasible. Though laparoscopic wedge resection is expected to be a preferable choice for GISTs compared with traditional open wedge resection as previously reported [27,29-33,35,36], more convincing evidence is still needed to prove its safety and feasibility. This manuscript summarizes the outcomes of LWR of gastric GISTs in the relatively larger series of patients to date. Our data demonstrate that the patients who underwent LWR for gastric GISTs results in effective control of the disease with minimal perioperative morbidity and no mortality. Besides, a rapid systematic review with a meta-analysis was conducted to summarize all the published information. We believe this could help surgeons to share the optimal individualized decision for patients.

Our series of patients who underwent LWR had less intraoperative blooding than that in the OWR group. The reduced length of incision wound, accurate operation and the application of energy-dividing devices, contributed to the reduction of blood loss. Pain after surgery was milder in LWR than in OWR, reflecting as the shorter duration or the lower dosage of analgesic application [29,32]. The time to first flatus was also earlier in LWR than in OWR, which indicated more rapid recovery of gastrointestinal function after LWR. Reduced use of analgesic drugs, shortened time of abdominal cavity exposure, alleviated inflammatory reactions, and earlier postoperative activities are considered to be the main reasons for earlier gastrointestinal recovery from LWR; all of which may also contribute to shortening the duration of postoperative hospital stay. The meta-analysis also revealed these mini-invasive advantages of the LWR. Interestingly, the operative time in the LWR group did not longer than OWR which is different from many other type of laparoscopic surgery [37-42]. This is because the time-consuming laparoscopic lymphadenectomy is unnecessary for GISTs resection due to the fact that the lymphatic metastasis of GISTs is quite rare. As time spending on the establishment of pneumoperitoneum and the closure of the trocar incision and minilaparotomy is likely to shorter than the open and closure of laparotomy, it is possible that the operative time for the LWR will be shorter than OWR with the development of the surgical techniques and laparoscopic instruments.

Regarding the postoperative complication, the incidence was higher in the OWR group than the LWR group, but the difference did not reach statistical significance in our study. However, the meta-analysis indicated a significant reduction in the LWR group (*P* = 0.04). In our study, there was a high incidence of wound problem in the OWR group. This is also true in included trials which specified the morbidity [31,34]. It was conceivable that complications other than wound problem were similar between groups because LWR results in the same organ and tissue resection as OWR.

LWR for gastric GISTs seems to have become a popular technique, the indications for this procedure in relation to tumor size are still controversial. Large lesions increase the difficulty to resect using endoscopic linear staplers and the risk of tumor spillage when removal. It was previously suggested by the NCCN Guidelines updated in 2007 for Optimal Management of Patients with GIST that laparoscopic techniques could be approached for tumors less than 5 cm [2]. However, many investigators have reported successful and safe removal of larger GISTs [26,43-45]. In our series, 12 cases with tumor size larger than 5 cm underwent LWR successfully with no conversion, demonstrating its feasibility, though the mean tumor size of OWR group was slightly larger than LWR group (4.3 versus 3.5 cm). This observation was mainly biased by the inherent selection process for patients to undergo a laparoscopic approach. patients with smaller tumors may be more amenable to laparoscopy versus larger tumors, which may tend to treat with laparoscopy or laparotomy. There were 20 cases in our series with tumor located near the esophagogastric junction or the pylorus who were considered inappropriate to undergo LWR. Performing this procedure has a high possibility of stenosis or deformity in the gastric inlet or outlet because it is associated with excessive resection of the healthy tissue of the gastric wall by laparoscopic stapling [46]. If the tumor was extraluminal growth, we resected the tumor using ultrasonic scalpel, then used laparoscopic intracorporeal handsewn method to close the incision. With those intraluminal growth, the laparoscopic transgastric wedge resection, which provided direct vision of the lesion and inner stomach, and allows better control of the surgical margin, was introduced. Whereas, if the tumor was large, laparoscopic distal or total gastrectomy was performed instead of LWR which is impossible in such situation. After tumors removal, all these cases were confirmed by intraoperative gastroscope examination to avoid gastric inlet or outlet narrowing. Currently, there are still no clear consensus guidelines for gastric GISTs laparoscopic approach based on tumor size and location. We advocate that tumor size and location should not be an absolute contraindication to laparoscopic techniques. However, one thing should be in mind that regardless laparoscopy or open surgery, it must be avoid direct tumor manipulation in an effort to eliminate the incidence of tumor rupture since tumor spillage can results in shortened disease-free survival [47].

With recent trials confirming the short-term surgical safety and long-term survival efficacy of laparoscopic techniques in other gastrointestinal malignancy [48-53], the role of laparoscopic surgery in resection of GISTs of the stomach should be clarified. Long-term survival remain critical for all patients with GISTs regardless of a benign or malignant designation since these tumors have an uncertain biologic behavior. It is widely accepted that the tumor size and mitotic index are two key factors on GISTs long-term outcomes. Several recent reports have also detailed recurrence rates of patients with gastric GISTs after laparoscopic surgery ranging from 4.8 to 18% [11,26,29,54,55]. In our study, LWR group had a lower recurrence rate (1.1%) compared to previous reports. This result was mainly due to most of patients in LWR group with tumors at very low, or low malignant risk (73.1%), whose recurrence may be delayed for as long as 10 years [56]. Moreover, in our center patients with tumors at moderate, or high malignant risk are routinely recommended to continue treatment with imatinib after surgery, which can effectively improved recurrence-free survival and overall survival of GIST patients with a high risk of GIST recurrence [57,58]. Despite the fact that tumor size of OWR group was larger than LWR group, two groups were comparable with no significant difference according to Fletcher’s criteria. Our series demonstrates the oncologic safety of the laparoscopic approach, with efficacy and recurrence rates similar to open surgical controls. All the tumor-recurrent cases in our study developed recurrence in 2 years after surgery, once more proving that most of the GISTs recurrence occurs within the first 2 years after surgery [3,11,59].

As nonadenocarcinomas in the stomach are uncommon, the sample of 156 patients is considered large. But it is still small for definitive conclusions on the safety and effectiveness of LWR. Thus, our rapid systematic review and meta-analysis synthesized the existing observational studies with strictly limiting inclusion and exclusion criteria. The included studies were primarily derived from the countries with the most widespread use of laparoscopic gastrectomy (two from Japan, one from Korea, one from China, one from Singapore, and one from Italy), and the total number of cases incorporated in the study was 525. The larger the number of patients in a meta-analysis, the greater its power to detect a possible treatment effect. Therefore, our comprehensive meta-analysis will contribute to a more systematic and objective evaluation for the safety and effectiveness of LWR.

There are some limitations to our study. The major bias was derived from retrospective nature and lack of prospectively defined inclusion criteria for those undergoing LWR. Also, the majority of cases in our study are in the past 3 years, which is short for the low risk GISTs to develop recurrence, and the follow up will continue. Although the meta-analysis confirmed the mini-invasive benefits of LWR and the similar postoperative and oncological outcomes between LWR and OWR, the simple size of some articles included was quite small and there was no prospective or randomized study that can markedly undermine the strength of the analysis. Our results should be confirmed by further randomized controlled trials that compare the open versus laparoscopic approach for the treatment GISTs.

## Conclusions

This study demonstrates laparoscopic wedge resection is a technically and oncologically safe and feasible approach for GISTs compared with open wedge resection. Moreover, laparoscopic wedge resection appears to be a preferable choice with mini-invasive benefits based on our data and a rapid systematic review with a meta-analysis.

## Abbreviations

LWR: Laparoscopic wedge resection
OWR: Open wedge resection
GISTs: Gastrointestinal stromal tumors
RR: Risk ratio
WMD: Weighted mean differences
CI: Confidence intervals
NOS: Newcastle-Ottawa Quality Assessment Scale
SD: Standard deviation
RFS: Relapse-free survival
HPFs: High-power fields
SMA: Smooth muscle actin
BMI: Body mass index
